# Overcoming chemical barriers: a new species of *Rhabdias* (Nematoda: Rhabdiasidae) from *Dendrobates tinctorius* (Anura: Dendrobatidae) in the Brazilian Amazon

**DOI:** 10.1017/S003118202510108X

**Published:** 2026-01

**Authors:** Lorena Freitas Souza Tavares-Costa, Talita Pantoja Ribeiro, Ronald Ferreira Jesus, Fred Haick, Maria Isabel Müller, Francisco Tiago Vasconcelos Vasconcelos Melo

**Affiliations:** 1 Laboratory of Cellular Biology and Helminthology “Profa. Dra. Reinalda Marisa Lanfredi”, Institute of Biological Sciences, Federal University of Pará (UFPA), Belém, Brazil; 2 Vale Institute of Technology (ITV), Belém, Brazil; 3 Department of Microbiology, Oregon State University (OSU), Corvallis, USA; 4 Department of Ecology and Evolutionary Biology, Federal University of São Paulo (Unifesp), São Paulo, Brazil

**Keywords:** anuran, molecular, nematoda, neotropical forest, taxonomy

## Abstract

The nematode genus *Rhabdias* comprises over 100 species of parasitic nematodes that infect amphibians and reptiles, with a wide geographical distribution. To date, 25 species have been reported from the Neotropical region. Despite this diversity, few integrative studies, combining morphological and molecular data have been conducted to characterize species within the genus. Therefore, the main objective of the present study is to describe, through an integrative approach, a new species of *Rhabdias* found parasitizing the lungs of an anuran with a high concentration of skin toxins, *Dendrobates tinctorius*, from the Brazilian Amazon. The new species of *Rhabdias* is characterized by an elongated body, uniform cuticular inflation attenuated at the extremities, 4 submedian lips and 2 lateral lips, a cup-shaped buccal capsule, and an elongated tail. The morphology of the buccal capsule in *Rhabdias camposi* n. sp. is also unique among *Rhabdias* representatives, as this morphological character is known so far. Thus, we emphasize that a detailed study of this morphological trait for species of the genus will be crucial for species diagnosis. Molecular and phylogenetic analyses were performed using mitochondrial COI gene sequences. We observed that the new taxon is closely related to *Rhabdias waiapi*, a parasite of *Pristimantis chiastonotus. Rhabdias camposi* n. sp. represents the 26th species of the genus reported from the Neotropics in amphibians and the first described from a *Dendrobates tinctorius* host in Brazil.

## Graphical abstract

**Figure:**
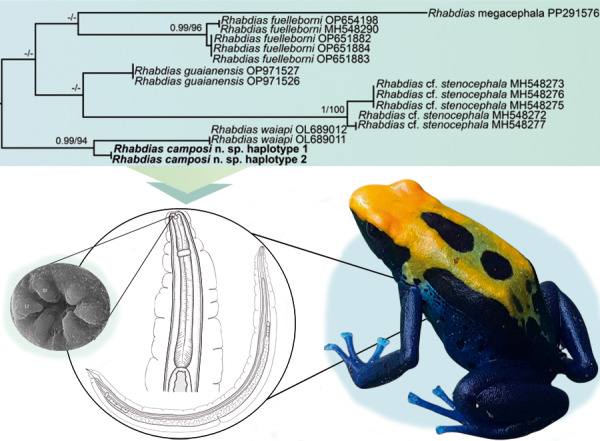

## Introduction

Nematodes of the genus *Rhabdias* are lung-dwelling parasites of amphibians and some reptiles. Amphibians become infected when the parasites actively penetrate their skin, while reptiles are infected through oral transmission (Kuzmin and Tkach, 2025). These parasites have a monoxenous life cycle, comprising a parasitic generation of hermaphroditic females and a free-living generation with separate male and female adults (Kuzmin, 2013; Langford and Janovy, 2013). *Rhabdias* was the first genus described within the family Rhabdiasidae and currently includes approximately 100 species with a global distribution (Kuzmin and Tkach, 2025).

Twenty five species of *Rhabdias* have been described from the Neotropical region (Alcantara et al., 2023; Euclydes et al., 2024), of which 15 occur in Brazil: *Rhabdias androgyna* (Kloss, 1971); *R. breviensis* (Nascimento et al., 2013); *R. elegans* (Kloss, 1971); *R. fuelleborni* (Travassos, 1926); *R. galactonoti* (Kuzmin et al., 2016); *R. glaurungi* (Willkens et al., 2019); *R. guaianensis* (Alcantara et al., 2023); *R. hermaphrodita* (Kloss, 1971), *R. matogrossensis* (Alcantara et al., 2023); *R. megacephala* (Euclydes et al., 2024); *R. paraensis* (Santos et al., 2011); *R. pocoto* (Morais et al., 2020); *R. pseudosphaerocephala* (Kuzmin et al., 2007); *R. stenocephala* (Kuzmin et al., 2016) and *R. waiapi* (Tavares-Costa et al., 2022).

Integrating molecular and morphological approaches provides crucial data for taxonomic descriptions. However, few studies have used integrative methods for the genus *Rhabdias* (Tkach and Snyder, 2014; Müller et al., 2018; Morais et al., 2020). Among the 15 species reported in Brazil, only 9 have been analysed using such approaches: *R. breviensis, R. fuelleborni, R. glaurungi, R. guaianensis, R. matogrossensis, R. megacephala, R. pocoto, R. pseudosphaerocephala,* and *R. waiapi*.

*Dendrobates tinctorius* (Cuvier, 1797) is an anuran species from the family Dendrobatidae that lives in the dense, humid tropical forests of the eastern Guiana Shield. Its range of distribution includes southern southeastern Guyana, northern Brazil, French Guiana, and southern Suriname (Rojas and Pašukonis, 2019; Frost, 2025). The species is diurnal and terrestrial, and feeds mainly on insects and small arachnids (Rojas and Pašukonis, 2019; Moskowitz et al., 2022). Despite researchers having studied this species due to its high toxicity (Moskowitz et al., 2022), there are still no reports on its helminth fauna.

During a biodiversity survey of amphibian helminth fauna in the state of Amapá, we identified nematodes belonging to the genus *Rhabdias*, characterized by morphological features distinct from those of known species. Based on morphological, molecular, and phylogenetic analyses of mitochondrial COI gene, we propose that these nematodes represent a new species.

## Materials and methods

### Host collection and morphological study of parasites

During a survey of the parasitic fauna of anurans in the ‘Beija-flor Brilho de Fogo’ Extractive Reserve, located in Pedra Branca do Amapari municipality, state of Amapá, Brazil (0°47′30·6″N, 51°58′42 1″W) in September 2021, 29 specimens of *D. tinctorius* were manually captured through an active visual search. After collection, the specimens were anesthetized with 2% lidocaine hydrochloride (CFMV, 2013), measured, weighed, and necropsied. The internal organs were removed, dissected, and analysed under a stereomicroscope. The nematodes found in the lungs were collected, cleaned in saline solution, sacrificed in heated 70% ethanol, and preserved in microtubes containing 70% ethanol at room temperature.

For morphological and morphometric analyses, the nematodes were clarified in Aman’s lactophenol 20%, mounted on temporary slides, and observed using an Olympus BX41 (Olympus, Tokyo, Japan) microscope equipped with a camera lucida for drawings and take measurements. The illustrations were prepared using CorelDRAW 2024 and processed with Adobe Photoshop 21.0.2. Prevalence and mean intensity were calculated according to Bush et al., (1997). The morphological measurements of the specimens are presented as the values of the holotype, followed by the mean of the paratypes and range in parentheses (reported in micrometres, except where indicated), following the standardization proposed by Willkens et al., (2019).

Three specimens were prepared for scanning electron microscopy (SEM) to examine external ultrastructural features. The nematodes were post-fixed in 1% osmium tetroxide (OsO_4_), dehydrated through a graded ethanol series (30–100%), dried using a CO₂ critical point dryer, mounted on aluminium stubs with carbon tape, and sputter-coated with gold/palladium. The specimens were then examined using a Vega3 scanning electron microscope (TESCAN, Brno, Czech Republic) operating at 10–20 kV in the Laboratory of Cellular Structural Biology (LBE) at the Federal University of Pará (UFPA).

### Molecular analyses and phylogenetic study

For molecular analysis, nematode specimens were placed in microtubes with absolute ethanol (100%) and stored at −20°C for preservation. To verify specimen identity, the anterior and posterior regions were separated and stored individually in ethanol, while the central region of the body was designated for DNA analysis. The hologenophore, following Pleijel et al., (2008), was preserved and deposited in a helminthological reference collection as a voucher specimen. Genomic DNA extraction utilized 200 µL of a 5% Chelex® solution (prepared in deionized water) and 2 µL of proteinase K, adhering to the manufacturer’s instructions. Samples were incubated at 56 °C for 14 hours, followed by heating at 90 °C for 8 minutes and centrifugation at 14 000 rpm for 10 minutes.

A partial fragment of the mitochondrial cytochrome c oxidase subunit I (COI) gene was amplified by PCR using specific primers (Forward primer *Rhabdias* COI1F 5’-GGKTTTTTTATGGGTAAYGGTC-3’ and reverse primer *Rhabdias* COIR 5’-GCNCCAGCYAANACWGGNAAAG-3’) described by Müller et al., (2018) and thermal cycling conditions described by Müller et al., (2018). The PCR products were visualized on 1% agarose gels to assess amplification success and fragment size, then purified using the QIAquick PCR Purification Kit (Qiagen®).

The purified products were sequenced using the Big Dye® Terminator v3.1 Cycle Sequencing Kit on an ABI 3730 DNA Analyzer at the Human Genome and Stem Cell Research Center, Institute of Biosciences, University of São Paulo. The resulting sequences were assembled with Sequencher v.5.2.4 and compared to reference sequences in the NCBI database using the BLASTn tool.

For the outgroup in the phylogenetic analysis, the species *Serpentirhabdias fuscovenosa* (Railliet, 1899) (accession number MH281971) and *Serpentirhabdias atroxi (*Kuzmin et al., 2016) (MH281969) were selected. Sequence alignment was performed using MUSCLE (Edgar, 2004), with the default settings in Geneious 7.1.3 software. After alignment, terminal regions were trimmed, and the presence of stop codons was checked using the second translation frame for invertebrate mitochondrial code, also in Geneious 7.1.3 (Kearse et al., 2012).

To evaluate substitution saturation in the aligned dataset, the Iss index was calculated using DAMBE 7 (Xia, 2018). Estimates of genetic divergence, including pairwise base substitution rates per site, were obtained in MEGA11 (Kimura, 1980; Tamura et al., 2021), with standard errors generated through a bootstrap procedure with 1000 replicates. The best nucleotide substitution model was identified as GTR + I + G, based on the Akaike Information Criterion (AIC) using jModelTest (Posada, 2008), and this model was subsequently used in the phylogenetic reconstructions.

Phylogenetic reconstructions were performed using Maximum Likelihood (ML) in RAxML 8.2.12 and Bayesian Inference (BI) in MrBayes 3.2.7a software, respectively (Guindon and Gascuel, 2003; Ronquist et al., 2003). Both analyses were conducted in CIPRES Science Gateway (Miller et al., 2010). Maximum likelihood inference (ML) was performed using bootstrap support values of 1000 repetitions, and only nodes with a bootstrap percentage (BP) greater than 70% were considered well-supported.

Bayesian analyses employed the following settings for the dataset: Iset nst = 6, rates = invgamma, ngammacat = 4, nucmodel = 4by4, code = universal, prset statefreqpr = dirichlet (1,1,1,1). For the Markov Chain Monte Carlo (MCMC), search chains were run with 10 000 000 generations, saving 1 tree every 1500 generations. The first 25 000 generations were discarded on the burn-in. The consensus tree (majority rule) was estimated using the remaining topologies, and we added commands sumt relburnin = yes, and sump relburnin = yes. Only nodes with Bayesian posterior probabilities greater than 90% were considered well-supported. The trees were visualised and edited in the software FigTree v1.3.3 (Rambaut, 2009).

## Results

### Systematics

Family: Rhabdiasidae Railliet, 1915

Genus: *Rhabdias* Stiles & Hassall, 1905

Species: *Rhabdias camposi* n. sp. Tavares-Costa and Melo

### Taxonomic summary

Type host: *Dendrobates tinctorius* (Cuvier, 1797) (Amphibia: Dendrobatidae)

Type locality: Beija-Flor Brilho de Fogo Extractive Reserve, Pedra Branca do Amapari municipality, state of Amapá, Brazil (0°47′30·6″N, 51°58′42·1″W).

Site of infection: Lungs.

Numbers of specimens/hosts, prevalence, mean infection intensity, and range: A total of 24 nematodes were found in 29 frogs, *P* = 51·7%; 1·6 (1–5).

Type material: One holotype and 11 paratypes were deposited at the Museum Emílio Goeldi, under numbers: Holotype: (MPEG 000324), hologenophore (MPEG 000323) and paratypes: (MPEG 000325).

GenBank Accession number: PX509145 and PX509146

ZooBank registration:The Life Science Identifier for *R. camposi* n. sp. is urn:lsid:zoobank.org:act:AB0746D2-66E4-4965-A2DA-AE5465DB0B3F

*Etymology*: The specific epithet *camposi* is dedicated to Professor Dr. Carlos Eduardo Costa Campos, in recognition of his invaluable contributions to studies of the Amazonian herpetofauna. During his lifetime, Dr. Carlos Eduardo played a fundamental role in the development of this and many other works, leaving a lasting legacy in adivancing zoological research in the Brazilian Amazon.

### Description

**Figure 1.**
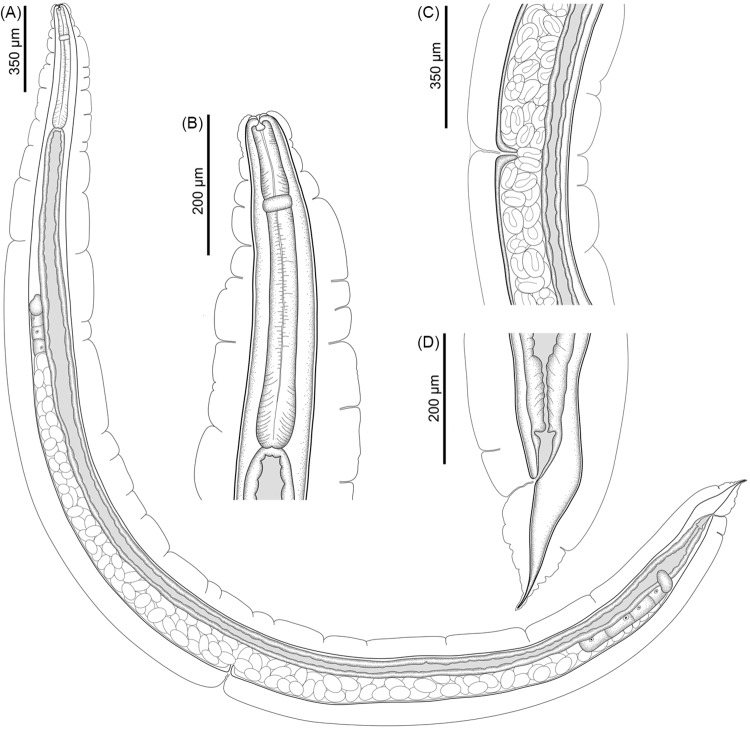
Line drawings of *Rhabdias camposi* n. sp. from *Dendrobates tinctorius.* (A) Entire body, lateral view; (B) anterior end of the body, lateral view; (C) vulva region, lateral view; (D) caudal end, lateral view.

**Figure 2.**
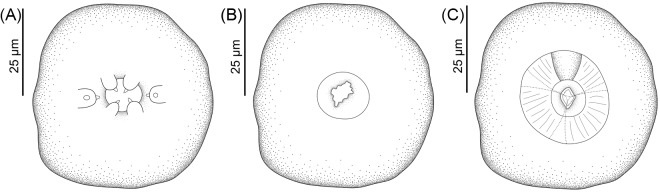
Line drawings of cross sections of anterior end and face view of *Rhabdias camposi* n. sp. from *Dendrobates tinctorius*. (A) Anterior extremity end face view; (B) optical section through anterior part of buccal capsule; (C) optical section through posterior part of buccal capsule.

**Figure 3.**
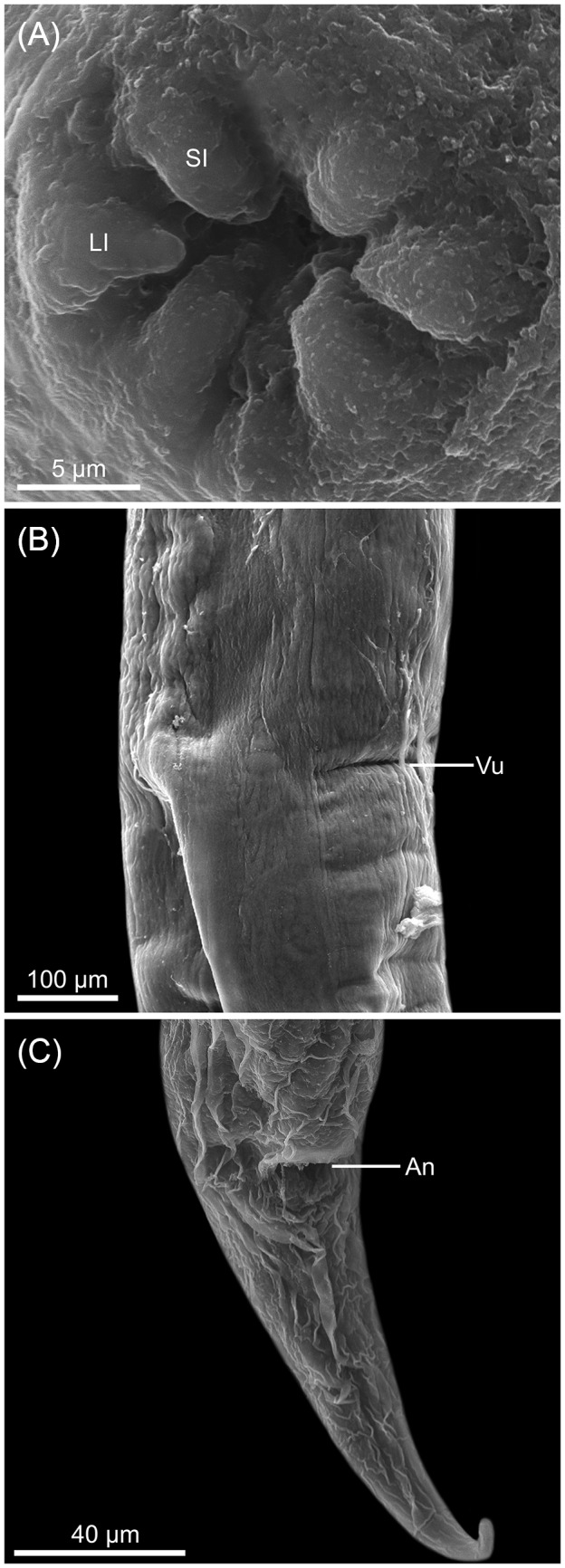
Scanning electron micrographs of *Rhabdias camposi* n. sp. from *Dendrobates tinctorius*. (A) Apical view (Ll – lateral lips; Sl – submedian lips); (B) mid-body region with partially latero-ventral view of the vulva (Vu – vulva); (C) posterior end, ventro-lateral view (An – anus).

See Figures 1-3 and Table 1 (based on the holotype and 11 paratypes, all gravid hermaphrodites). Body slender, elongated, 4·4; 4·6 (3·7–5·3) mm. Body surface covered by prominent cuticular inflation, uniform along body and attenuated at extremities (Fig. 1A). Lateral pores and ducts present along body length. Body width at vulva 188; 217 (158–258), width at oesophagus-intestine junction 108; 110 (101–123). Oral opening, round in shape, surrounded by 6 lips, 4 lips, close to the edge of the oral opening and projecting inward of oral opening (toward the oral lumen), and 2 larger lateral lips, located farther from opening (Figs 2A, 3A). Each lip bears a papilla on its inner edge, amphids located posterior to lateral lips (Fig. 2A). Buccal capsule cup-shaped with 10; 9 (8–10) deep and 14; 14 (13–16) wide, depth/width ratio 0·7; 0·68 (0·6–0·7). Buccal capsule walls consist of larger anterior portion and smaller posterior portion, both with irregular folds on internal surface of wall (Fig. 2B). Buccal capsule close to entrance of oesophagus lumen with serrated internal surface and squared margins. Entrance of oesophageal lumen triangular, with rounded edges and oesophageal gland, located in the dorsal region of oesophagus (Fig. 2C). Oesophagus length 467; 484 (427–509), representing 11%; 10% (9–12%) of body length, claviform with rounded apex and with distinctly rounded dilation at anterior region (Fig. 1B). Width at anterior end of oesophagus 29; 31 (28–33), width at anterior dilation of oesophagus 34; 38 (35–40), width after dilation 32; 36 (34–37), bulb width 53; 59 (53–64). Nerve-ring surrounding oesophagus posterior to dilation located at 151; 154 (134–168) from anterior end (Fig. 1B). Excretory pore not observed. Genital system typical of Rhabdiasidae, amphidelphic with anterior and posterior ovaries, transverse vagina, post-equatorial vulva, located at 2·4; 2·5 (2·1–2·9) mm from anterior extremity (representing 55%; 54% (51–56%) of the body length). Vulvar lips slightly protruding (Fig. 3B). Uterus thin-walled, with numerous eggs (> 100), embryonated eggs close to vulva. Egg size 76; 80 (75–85) × 43; 45 (39–49) (*N* = 10 eggs measured from uterus of holotype and each paratype) (Fig. 1C). Female reproductive system flexed in ‘U’ shape at 1·100; 1273 (987–1447) from anterior extremity (near oesophagus–intestine junction) and 585; 613 (561–647) of posterior region (close to rectum). Intestine thick-walled. Rectum short, funnel-shaped, and lined with thin cuticle (Fig. 1D). Contents of intestine brown throughout length. Tail long, gradually tapering 187; 210 (177–251) and 4·3%; 5% (3·7–5·3%) of body length (Fig. 3C).

**Table 1.** Selected morphological characters of *Rhabdias* spp. From the Neotropical region. Morphological measurements are given in micrometres, unless otherwise indicated. Bold indicates the new species. Abbreviations: BL = body length; BWV = body width at vulva; NL = number of lips; MDBC = maximum diameter of the buccal capsule; TDBC = total depth of the buccal capsule; OL = oesophagus length; NR = nerve ring; VP = vulva position

Species Neotropical Region	Type Host	Country	BL (mm)	NL	MDBC	TDBC	OL	NR	VP	Tail	Reference
***Rhabdias camposi*** n. sp.	*Dendrobates tinctorius* (Cuvier, 1797)	Brazil	3·7−5·3	6	13−16	8−10	427−509	134−168	Post equatorial	177−251	This study
*Rhabdias alabialis*	*Rhinella marina* (Linnaeus, 1758)	Costa Rica	7·67−9·27	Absent	10−15	10	340−445	136−167	Equatorial	278−328	Kuzmin et al., 2007
*Rhabdias androgyna*	*Rhinella* cf. *margaritifera* (Laurenti, 1768)	Brazil	7·9−15·4	6	19−27	7−9	598−751	210−300	Pre equatorial	304−455	Kloss 1971
											Kuzmin et al., 2015
*Rhabdias anolis*	*Anolis frenatus* Cope, 1899	Panama	4·6−7·4	6	9−13	9−13	323−360	129−144	Pre equatorial	242−306	Bursey et al., 2003
*Rhabdias breviensis*	*Leptodactylus petersii* (Steindachner, 1864)	Brazil	2·63−3·63	6	7−13	4−9	238−410	41−84	Post equatorial	139−191	Nascimento et al., 2013
*Rhabdias elegans*	*Rhinella arenarum* (Hensel, 1867)	Argentina	4·55−9·5	Absent	7	7	314−490	450−860	Slightly post equatorial	255−400	Kloss 1974
*Rhabdias fuelleborni*	*Rhinella marina* (Linnaeus, 1758)	Brazil	6·2−12·7	6	13−19	6−9	355−552	156−255	Pre equatorial	313−586	Kuzmin et al., 2015
											Müller et al., 2023
*Rhabdias galactonoti*	*Adelphobates galactonotus* (Steindachner, 1864)	Brazil	4·9−7·2	6	14−20	10−12	349−487	149−226	Pre equatorial	229−353	Kuzmin et al., 2016
*Rhabdias glaurungi*	*Scinax* gr *ruber* (Laurenti, 1768)	Brazil	3·54−6·73	6	10−16	6−9	292−332	108−140	Post equatorial	151−216	Willkens et al., 2019
*Rhabdias guaianensis*	*Leptodactylus podicipinus* (Cope, 1862)	Brazil	2·9−3·5	4 submedian lips and 2 lateral pseudolabia	13−14	11−14	267−275	113−114	Post equatorial	160−187	Alcantara et al., 2023
*Rhabdias hermaphrodita*	*Rhinella crucifer* (Wied-Neuwied, 1821)	Brazil	4−12	–	–	–	268−663	–	Equatorial	226−524	Kloss 1971
*Rhabdias kuzmini*	*Incilius occidentalis* (Camerano, 1879)	Mexico	14·15−19·19	4 submedian lips and 2 lateral pseudolabia	35−47	31−70	833−1008	302−407	Equatorial vulva or post equatorial	271−380	Martinez-Salazar and Leon-Regagnon 2007
*Rhabdias leonae*	*Anolis megapholidotus* Smith, 1933	Mexico	11·44−15·06	4 submedian lips and 2 lateral pseudolabia	23−34	11−19	670−751	217−244	Post equatorial	217−325	Martinez-Salazar 2006
*Rhabdias manantlanenesis*	*Craugastor occidentalis* (Taylor, 1941)	Mexico	6·48−9·64	4 submedian lips and 2 lateral pseudolabia	19−27	11−19	387−515	193−244	Pre equatorial	143−232	Martinez-Salazar 2008
*Rhabdias matogrosensis*	*Leptodactylus podicipinus (Cope, 1862)*	Brazil	2·9−3·8	4 submedian lips and 2 lateral pseudolabia	15−17	11−16	437−508	142−208	Post equatorial	166−230	Alcantara et al., 2023
*Rhabdias megacephala*	*Proceratophrys boiei* (Wied-Neuwied, 1824)	Brazil	8·1−9·9	4	22 – 34	11−23	630−799	152−503	Post equatorial	244−338	Euclydes et al., 2024
*Rhabdias nicaraguensis*	*Anolis capito* Peters, 1863	Nicaragua	6·6−7·2	6	11−15	11−15	536−612	134−159	Equatorial	255−357	Bursey et al., 2007
*Rhabdias paraensis*	*Rhinella marina* (Linnaeus, 1758)	Brazil	5·92−8·19	Absent	3·9−16·8	14·9−27·9	395−440	151−234	Post equatorial	266−346	Santos et al., 2011
*Rhabdias pocoto*	*Pseudopaludicola pocoto* Magalhães, Loebmann, Kokubum, Haddad, and Garda, 2014	Brazil	3·41−7·43	6	11−17	9−17	475−677	238−339	Equatorial	98−163	Morais et al., 2020
*Rhabdias pseudosphaerocephala*	*Rhinella marina* (Linnaeus, 1758)	Nicaragua	6·17−9·60	4 submedian lips and 2 lateral pseudolabia	15−17	7−12	400−46	140−190	Equatorial	310−410	Kuzmin et al., 2007
*Rhabdias savagei*	*Lithobates forreri* (Boulenger, 1883)	Costa Rica	4·2−5·3	4	18−24	12−18	366−415	116−134	Post equatorial	171−201	Bursey and Goldberg 2005
*Rhabdias stenocephala*	*Leptodactylus pentadactylus* (Laurenti, 1768) *Leptodactylus paraensis* Heyer, 2005	Brazil	6·9−8·1	6	15−18	8−11	354−453	168−225	Post equatorial	234−345	Kuzmin et al., 2016
*Rhabdias tobagoensis*	*Pristimantis incertus* (Lutz, 1927)	Tobago Island	7·34−7·56	6	18−21	6−9	476−530	177	Equatorial (slightly pre or post equatorial)	210−276	Moravec and Kaiser 1995
											Willkens et al., 2019
*Rhabdias waiapi*	*Pristimantis chiastonotus* (Lynch and Hoogmoed, 1977)	Brazil	3·36−5·38	6	16−19	8	405−533	145−187	Post equatorial	173−274	Tavares-Costa et al., 2022

### Remarks

The new species was assigned to the genus *Rhabdias* based on molecular data and the following morphological characteristics: inflated body cuticle, a cup-shaped buccal capsule, amphidelphic reproductive system with a short transverse vagina, morphology of the oral opening, and tail morphology. Additionally, it was found parasitizing the lungs of anurans. According to Kuzmin (2013), Müller et al., (2018) and Tavares-Costa et al., (2022), those morphological traits are the main characteristics for this genus.

*Rhabdias camposi* n. sp. differs from other species reported in the Neotropical region by a unique combination of morphological features, including the position of the nerve ring, dimensions of the buccal capsule, shape of the cuticular inflation, oesophagus length, lip position and size, and tail length. Therefore, based on the distinctive set of morphological traits observed in *Rhabdias camposi* n. sp., we propose it as a new species.

We compared this new taxon with *Rhabdias* species reported in Neotropical anurans (Table 1). However, as suggested by Willkens et al., (2019), *Rhabdias mucronata* Schuurmans-Stekhoven, 1952 and *Rhabdias truncata* Schuurmans-Stekhoven, 1952 are excluded from the comparison, as morphological data for their mature forms are unavailable and their descriptions were based on juvenile specimens found in the host’s body cavity. In addition, we compare the new taxon with *R. hermaphrodita* (Kloss, 1971), a Neotropical species for which the original description lacks information regarding the arrangement of the oral structures.

Based on the morphology and arrangement of the oral opening structures, *Rhabdias camposi* n. sp. is part of the group of Netropic species that have 6 lips (4 submedial and 2 lateral). This group is composed of 10 species: *Rhabdias androgyna* (Kloss, 1971); *R. breviensis* (Nascimento et al., 2013); *R. fuelleborni* (Travassos, 1926); *R. galactonoti* (Kuzmin et al., 2016); *R. glaurungi* (Willkens et al., 2019); *R. manantlanensis* (Martinez-Salazar, 2008); *R. pocoto* (Morais et al., 2020); *R. stenocephala* (Kuzmin et al., 2016); *R. tobagoensis* (Moravec and Kaiser, 1995) and *R. waiapi* (Tavares-Costa et al., 2022) (Table 1).

*Rhabdias androgyna* (Kloss, 1971) was described in *Rhinella* gr. *margaritifera* (Laurenti, 1768) (Bufonidae) differ from the new species by the total length (7·9–15 mm *R. androgyna νs.* 3·36–5·38 mm *Rhabdias camposi* n. sp.), by morphology of the buccal capsule, in *R. camposi* n. sp., the capsule walls consist of a larger anterior portion and a smaller posterior portion, both of which display irregular folds on the inner surface. In contrast, *R. androgyna* lacks a defined posterior segment and exhibits a serrated inner wall along almost its entire length, except at the base (Kuzmin et al., 2015). Additionally, *R. androgyna* has a wider oral capsule (19–27 *R. androgyna νs* 13-16 *R. camposi* n. sp.) and longer oesophagus (598–751 *R. androgyna vs.* 427-509 *R. camposi* n. sp.). Position of the vulva also differs, *R. androgyna* has a pre-equatorial vulva located 3·7–7·2 mm from the anterior end (44·5–50·8% of the body length), while the new species has a post-equatorial vulva located 2·1–2·9 mm from the anterior end (51–56% of the body length). In addition, the anterior region of the *R. androgyna* body has a characteristic shape, which consists of a rounded dilation of the inflation with 2 layers, with the innermost layer connecting to the body wall in a body dilation like a ‘shoulder’ (Kloss, 1971; Kuzmin et al., 2015); this characteristic is not observed in *R. camposi* n. sp.

In comparison to the new species, *Rhabdias breviensis (*Nascimento et al., 2013) described in *Leptodactylus petersii* (Steindachner, 1864) (Leptodactylidae), has a smaller and wider body (2·63–3·63 mm × 370–543 *R. breviensis νs.* 3·7–5·3 mm × 158–258 *R. camposi* n. sp.) and has a body curvated dorsally. In addition, *R. breviensis* has a smaller dimensions of oral capsule (7–13 × 4–9 *R. breviensis νs*. 8–10 × 13–16 *R. camposi* n. sp.), shorter oesophageal length (238–410 *R. breviensis vs.* 427–509 *R. camposi* n. sp.), and a shorter distance from the anterior end to the nerve ring (41–84 *R. breviensis νs*. 134–168 *R. camposi* n. sp.). The vulva in *R. breviensis* is similar to that in the new taxon (post-equatorial), but also differs because in the former species, the vulva is far posterior to the anterior end (65–71% of the body length *R. breviensis vs.* 51–56% of the body length *R. camposi* n. sp.) and the tail is shorter (139–191 *R. breviensis νs.* 177–251 *R. camposi* n. sp.) (Nascimento et al., 2013).

*Rhabdias fuelleborni* (Travassos, 1926), originally described in *Rhinella diptycha* (= *Bufo marinus*) (Schneider, 1799) (Bufonidae), is longer than the new taxon (6·1–12·7 mm *R. fuelleborni vs* 3·7–5·3 mm *R. camposi* n. sp. in total length), and has a buccal capsule morphology composed of a smooth anterior wall and a posterior wall with irregular folds (Müller et al., 2023), whereas in *R. camposi*, both walls of the capsule have irregular folds on their inner surface. Vulva equatorial 3·1–6·3 mm from the anterior end (49·4–52% of the body length in *R. fuelleborni vs.* 51–56% of the body length in *R. camposi* n. sp.), and a longer tail (454–586 *R. fuelleborni vs.* 177–251 *R. camposi* n. sp.) (Travassos, 1926; Kuzmin et al., 2015; Müller et al., 2023).

Although also described in a dendrobatid anuran, *Rhabdias galactonoti* (Kuzmin et al., 2016), from *Adelphobates galactonotus* (Steindachner, 1864) (Dendrobatidae), presents several morphological differences when compared to the new taxon. It has a prominent cuticular inflation along the body, has larger body dimensions (5·6–6·04 mm in *R. galactonoti vs.* 3·7–5·3 mm in *R. camposi* n. sp.), the buccal capsule walls exhibit regular folds on the anterior wall and a posterior part that is shallow, thin-walled, and surrounded by the apex of the oesophagus (Kuzmin et al., 2016). In *R. camposi*, both walls of the capsule display irregular folds on their inner surface. *R. galactonoti* has smaller relative proportions of the oesophagus (6·3–8·2% of body length in *R. galactonoti vs*. 9–12% in *R. tinctorii* n. sp.), and a greater distance from the anterior end to the nerve ring (184–226 in *R. galactonoti vs.* 134–168 in *R. tinctorii* n. sp.). Additionally, the position of the vulva also differs: *R. galactonoti* has a pre-equatorial vulva (representing 43–50% of body length) (Kuzmin et al., 2016), while the new species has a post-equatorial vulva (51–56% of body length).

The new species can be distinguished from *Rhabdias glaurungi* (Willkens et al., 2019), described from *Scinax* gr. *ruber* (Laurenti, 1768) (Hylidae), by the arrangement of the lips: in the new species, 6 lips are present, 4 situated at the edge of the oral opening and 2 lateral lips positioned farther apart, whereas in *R. glaurungi* all 6 lips are positioned close to the oral opening (Willkens et al., 2019). In addition, *R. glaurungi* exhibits the opposite pattern in buccal capsule morphology, consisting of a smooth anterior part wall and a posterior part wall with an irregularly folded inner surface, and has a smaller oesophagus (427–509 in *R. camposi* n. sp. *vs.* 292–332 in *R. glaurungi*) (Willkens et al., 2019).

The description of *Rhabdias hermaphrodita* (Kloss, 1971) from *Rhinella crucifer* (Wied-Neuwied, 1821) (= *Bufo crucifer*) (Bufonidae) is superficial and incomplete; the author did not provide any morphometric or morphologic information on the buccal capsule or apical structures, as discussed by Willkens et al., (2019). However, it is possible to differentiate the 2 species by body length, *R. hermaphrodita* measures up to 12 mm in total length, while the new species has 3·7–5·3 mm in total length. In addition, *R. hermaphrodita* does not present dilation of the cuticular inflation in the anterior region (Kloss, 1971), while the new species has a very characteristic anterior end cuticular inflation.

Compared to the new taxon, *Rhabdias manantlanensis* (Martinez-Salazar, 2008) of *Craugastor occidentalis* (Taylor, 1941) (Craugastoridae) has a longer body length (6·48–9·64 mm *R. manantlanensis νs*. 3·7–5·3 mm *R. camposi* n. sp.), larger bucal capsule diameter (19–27 *R. manantlanensis νs.* 13–16 *R. camposi* n. sp.), greater distance from the anterior end to the nerve ring (193–244 *R. manantlanensis νs.* 134–168 *R. camposi* n. sp.), slightly pre-equatorial vulva *vs*. post-equatorial vulva (41·66–51·59% of the body length in *R. manantlanensis νs.* 51–56% of the body length in *R. camposi* n. sp.), and shorter tail (143–232 or 1·5–3·3% of the body length in *R. manantlanensis νs.* 177–251 or 3·7–5·3% of the body length in *R. camposi* n. sp.) .

*Rhabdias pocoto (*Morais et al., 2020), a parasite of *Pseudopaludicola pocoto* (Magalhães, Loebmann, Kokubum, Haddad & Garda, 2014) (Leptodactylidae), differs from *R. camposi* n. sp. in several morphological and morphometric aspects. The former species is larger (3·41–7·43 mm in *R. pocoto vs.* 3·7–5·3 mm in *R. camposi* n. Sp. in total length), exhibits the opposite pattern in buccal capsule morphology, consisting of a smooth anterior part wall and a posterior part wall with an irregularly folded inner surface, and has a deeper buccal capsule (9–17 *R. pocoto vs.* 8-10 *R. camposi* n. sp.), a longer oesophagus (475–677 or 8·9–13·9% of body length *R. pocoto vs.* 427–509 or 9–12% *R. camposi* n. sp.) and a shorter tail (98–163 *R. pocoto vs.* 177–251 *R. camposi* n. sp.). Additionally, *R. pocoto* has 2 subapical lateral pores connected to an amorphous gland-like structure in the anterior region (Morais et al., 2020), which is absent in *R. camposi* n. sp.

*Rhabdias stenocephala (*Kuzmin et al., 2016), described from *Leptodactylus pentadactylus* (Laurenti, 1768) and *Leptodactylus paraensis* (Heyer, 2005) (Leptodactylidae), can be easily distinguished from the new species by the presence of a prominent anterior constriction of the body, followed by a sudden expansion of the body wall posterior to this region (Kuzmin et al., 2016), features not observed in *R. camposi* n. sp. Furthermore, compared with *R. stenocephala*, the new species differs in buccal capsule wall structure: in *R. stenocephala*, the anterior portion is transparent, whereas the posterior portion is denser, with a circular thickening clearly visible in apical view. In addition, *R. stenocephala* is larger (6·9–8·1 mm in *R. stenocephala vs.* 3·7–5·3 mm in *R. camposi* n. sp.) (Kuzmin et al., 2016).

The new species can be easily distinguished from *Rhabdias tobagoensis* (Moravec and Kaiser, 1995), parasite of *Pristimantis incertus* (Lutz, 1927) (= *Eleutherodactylus terraebolivaris*) (Craugastoridae), by having a smaller body (7·34–7·56 mm in *R. tobagoensis vs.* 3·7–5·3 mm in *R. camposi* n. sp.), a smaller buccal capsule (6–9 × 18–21 *R. tobagoensis vs.* 8–10 × 13–16 *R. camposi* n. sp.), and the position of the vulva, which is equatorial in *R. tobagoensis* (Moravec and Kaiser, 1995), while in the new species it is post-equatorial (representing 45–49% of body length in *R. tobagoensis vs.* 51%–56% representing of body length in *R. camposi* n. sp). In addition, Willkens et al., (2019) re-examined deposited paratypes and added new morphological data. According to the authors, the posterior end of the oesophagus in *R. tobagoensis* is distinctly flattened and even concave, whereas it is rounded in *R. camposi* n. sp. This latter feature was not mentioned in the original description of *R. tobagoensis* (Moravec and Kaiser, 1995).

*Rhabdias waiapi* (Tavares-Costa et al., 2022), from *Pristimantis chiastonotus* (Lynch & Hoogmoed 1977), differs from the new taxon in having a deeper, more prominent cup-shaped buccal capsule (16–19 *R. waiapi vs.* 13–16 *R. camposi* n. sp.) with the posterior part smooth (Tavares-Costa et al., 2022), whereas in *R. camposi* n. sp. it bears irregular folds. Moreover, the internal surface adjacent to the oesophageal entrance is serrated in *R. camposi*, while it is smooth in *R. waiapi*. Furthermore, *R. waiapi* has a greater width at the oesophageal–intestine junction (130–173 in *R. waiapi vs.* 101–123 in *R. camposi* n. sp.), and a greater distance from the anterior end to the nerve ring (145–187 in *R. waiapi vs.* 134–168 in *R. camposi* n. sp.) (Tavares-Costa et al., 2022).

The buccal capsule morphology of *Rhabdias camposi* n. sp. is distinctive, and the unique combination of its morphological and morphometric features constitutes the diagnostic character set that supports *R. camposi* n. sp. as a distinct taxon. The morphology of the buccal capsule has been used and represents one of the most informative traits for species identification and differentiation within the genus (Kuzmin, 2013). Although the morphology of the buccal capsule is not known for all *Rhabdias* species, we reinforce that a detailed analysis of this structure is essential for the accurate recognition and delimitation of *Rhabdias* spp.

Additional morphological and metrical differences between the new species and *Rhabdias* spp. from the Neotropical realm can be observed in Table 1.

### Molecular analyses and phylogenetic study

Mitochondrial COI sequencing of *R. camposi* n. sp. resulted in 2 sequences of 345 bp (haplotype 1) and 338 bp (haplotype 2), while a BLASTn search revealed no identical (with 100% of identity) match with any other Rhabdiasid available in the NCBI database. The alignment of our sequences with those available in GenBank generated a matrix of 324 base pairs.

The Iss index indicated no saturation in the transitions or transversions; Iss.c values were greater than the Iss values. Pairwise genetic divergence comparison, considering *Rhabdias* spp. and *Serpentirhabdias fuscovenosa* (MH281971) and *Serpentirhabdias atroxi* (MH281969) (the outgroup for phyllogenetic analysis), revealed that *R. camposi* n. sp. has the lowest genetic differences with *R. waiapi* n. sp., (4% of genetic divergence) (see Supplementary Material Table 1).

**Figure 4.**
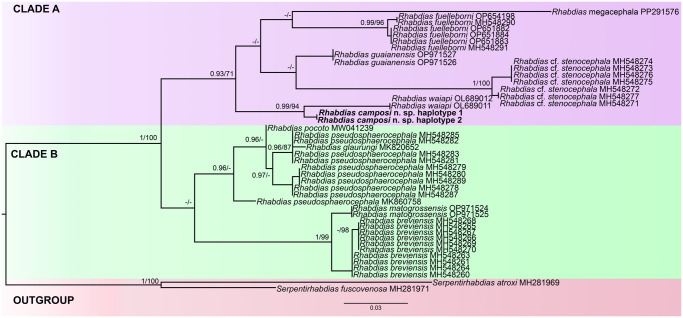
Maximum likelihood phylogenetic topology of *Rhabdias* spp. of COI gene using *Serpentirhabdias atroxi* and *Serpentirhabdias fuscovenosa* as outgroup, indicating the position of *Rhabdias camposi* n. sp. (represented in bold italics). GenBank accession numbers follow each taxon. Support values are above or below nodes: posterior probabilities <0·90 and bootstrap scores <70 are not shown, or are represented by a dash. Branch-length scale bar indicates number of substitutions per site.

Maximum-likelihood and Bayesian inference analyses based on 46 taxa revealed similar topologies. Two well-supported monophyletic lineages were consistently recovered, herein designated as Clade A and Clade B (Fig. 4).

Clade A comprises a strongly supported subclade grouping 2 sequences of *Rhabdias waiapi*, a parasite of *Pristimantis chiastonotus* (Lynch & Hoogmoed, 1977) (Strabomantidae), and 2 sequences of *Rhabdias camposi* n. sp., parasitic in *D. tinctorius* (Dendrobatidae). This subclade is, in turn, recovered as sister to a larger clade, which is divided into 2 subclades: the first includes sequences of *Rhabdias megacephala*, a parasite of *Proceratophrys boiei* (Wied-Neuwied, 1824) (Odontophrynidae) and *Rhabdias fuelleborni*, associated with *Rhinella* spp. (Bufonidae); the second subclade comprises *Rhabdias guaianensis*, a parasite of *Leptodactylus podicipinus* (Cope, 1862) and *Rhabdias cf. stenocephala*, parasitic in *Leptodactylus pentadactylus* (Laurenti, 1768), both members of the Leptodactylidae family (Fig. 4).

Clade B includes sequences of *Rhabdias breviensis* Nascimento, 2013, which are arranged into 2 well-defined subclades. This clade groups, also with strong nodal support, together with sequences of *Rhabdias matogrossensis*, a parasite of *Leptodactylus macrosternum* Miranda-Ribeiro, 1926 (all Leptodactylidae). In addition, phylogenetic analyses resolved *Rhabdias pseudosphaerocephala* (Kuzmin et al., 2007) into 2 well-supported and distinct clades: One comprising individuals from the northern, southeastern, and southern regions of Brazil, and another composed of individuals from the northeastern region (Fig. 4).

The latter clade formed a well-supported monophyletic group with *Rhabdias glaurungi*, a parasite of *Scinax* gr. *ruber* (Hylidae), and *Rhabdias pocoto*, parasitic in *Pseudopaludicola pocoto* Magalhães, Loebmann, Kokubum, Haddad & Garda, 2014 (Leptodactylidae).

## Discussion

*Rhabdias camposi* n. sp. represents the 26th species of *Rhabdias* described in the Neotropical region, the 15th in Brazil, and the second species recorded in amphibians from the state of Amapá, Brazil. The primary morphological features distinguishing the new taxon from its congeners include the morphology of cuticular inflation, arrangement and number of lips, buccal capsule dimensions, oesophagus length, and tail length.

The disposition, organization, and number of circumoral structures are key morphological traits for distinguishing species within the genus (Kuzmin, 2013; Kuzmin et al., 2024). In the Neotropical region, 25 species (23 of which parasitize amphibians) are recognized based on distinct oral arrangements, categorized into 4 main types: (1) 4 submedian lips and 2 lateral pseudolabia (*R. guaianensis, R. kuzmini, R. savagei, R. pseudosphaerocephala*); (2) 6 lips (*R. androgyna, R. breviensis, R. fuelleborni, R. galactonoti, R. glaurungi, R. manantlanensis, R. matogrossensis, R. nicaraguensis, R. pocoto, R. stenocephala, R. tobagoensis, R. waiapi*); (3) 4 lips (*R. leonae, R. megacephala, R. savagei*); and (4) absent lips (*R. alabialis, R. elegans, R. paraensis*) (Tavares-Costa et al., 2022; Alcantara et al., 2023; Euclydes et al., 2024). *R. mucronata, R. truncata* and *R. hermaphrodita* were excluded due to the lack of information on oral structure in their original descriptions (Willkens et al., 2019).

Despite belonging to the second group, *Rhabdias camposi* n. sp. can be distinguished from its congeners by a unique combination of morphometric and morphological features, including buccal capsule dimensions, morphology of both anterior and posterior portions of the buccal capsule, the shape of the cuticular inflation, oesophagus length, lip arrangement, and tail length. Furthermore, we found significant genetic divergence between the new taxon and sequences of *Rhabdias* species for which molecular data are available. Thus, the combination of morphology and molecular data supports the recognition of *R. camposi* n. sp. as a distinct taxon.

Although oral structures are widely used as diagnostic characters in *Rhabdias*, molecular evidence suggests that such features do not necessarily reflect monophyletic groupings (Tkach and Snyder, 2014; Müller et al., 2018; Tavares-Costa et al., 2022). Our data support this, demonstrating that closely related phylogenetic species can differ considerably in apical structures, whereas distantly related species may exhibit convergent morphologies.

Phylogenetic analyses placed *Rhabdias camposi* n. sp. in a strongly supported clade with *R. waiapi*, a species described by Tavares-Costa et al., (2022) using an integrative approach combining morphological and molecular data from specimens parasitizing *Pristimantis chiastonotus*. According to these authors, *R. waiapi* displays the second oral pattern (6 lips, 4 submedians, and 2 laterals), which was also observed in *Rhabdias camposi* n. sp. The morphological and phylogenetic affinities between the 2 species may be associated with the overlapping geographic distributions of their respective hosts, *P. chiastonotus* and *D. tinctorius*, which co-occur in the Guiana region and northern Brazil (Frost, 2025). Moreover, both nematode species were recorded in geographically close municipalities in Amapá state, Brazil.

These findings suggest sympatric distribution and a possible shared evolutionary origin between *R. waiapi* and *Rhabdias camposi* n. sp., potentially shaped by similar selective pressures in the Amazonian environment. Moravec and Sey (1990), Tkach and Snyder (2014) and Müller et al., (2018), also argued that parasite specificity and host distribution should be considered complementary parameters for evaluating taxonomic affinities within *Rhabdias*. The results of this study are in agreement with previous findings (Tkach and Snyder, 2014; Müller et al., 2018; Tavares-Costa et al., 2022; Alcantara et al., 2023; Euclydes et al., 2024) and reinforce the importance of integrative approaches combining morphological, molecular, and biogeographic data for robust species delimitation, especially among morphologically conserved or cryptic groups.

Phylogenetic and biogeographic studies of *Rhabdias* spp. have revealed cryptic diversity among Neotropical species, with 3 species complexes exhibiting high genetic diversity: *R. breviensis, R. pseudosphaerocephala,* and *R. stenocephala* (Müller et al., 2018, 2023). We also observed the same species complexes, even after including sequences of the new species. These findings support the hypotheses that host specificity within *Rhabdias* varies widely and is influenced by multiple ecological and evolutionary factors. Among these factors, ecological fitting deserves particular attention, as a central element in the establishment and maintenance of these associations, potentially allowing the progressive adaptation to novel hosts (Langford and Janovy, 2013).

We also found a well-supported monophyletic clade composed of sequences from *R. matogrossensis. R. breviensis* has been reported from various host families: Bufonidae, Odontophrynidae, Leptodactylidae, and Hylidae (Nascimento et al., 2013; Müller et al., 2018; Da Silva et al., 2019), while *R. matogrossensis* has only been found in leptodactylid hosts (Alcantara et al., 2023). These data suggest that the *R. breviensis* forms a complex of species that exhibits lower host specificity and was found infecting species from multiple anuran families.

Furthermore, we found that the genetic divergence between *R. breviensis* and *R. matogrossensis* ranged from 1·89% to 2·21%, indicating a close genetic relationship. Thus, we conclude that *R. matogrossensis* may represent a species that diverged recently within the *R. breviensis* complex. These findings support the existence of recently diverged cryptic species and reinforce the central role of ecological fitting in modulating host specificity (Langford and Janovy, 2013; Müller et al., 2018).

We recovered a monophyletic group (with low support) composed of 2 subclades: one formed by *R. fuelleborni*, parasitic in *Rhinella* spp. and *R. megacephala*, parasitic in *Proceratophrys boiei*; the other comprising *R.* cf. *stenocephala*, parasitic in *Leptodactylus* spp., and *R. guaianensis*, parasitic in *Leptodactylus podicipinus* (Cope, 1862). These results support the hypothesis raised by Müller et al., (2023), who suggested that *R. fuelleborni* may represent a distinct lineage within the *R.* cf. *stenocephala* complex, found in the Caatinga biome, that resulted from ecological fitting. Furthermore, *R.* cf. *stenocephala, R. fuelleborni,* and *R. megacephala* exhibit unique morphological traits, which are absent in other congeners. These morphological characters potentially represent characters that have some phylogenetic signal and are important for understanding the evolutionary patterns of this group. For instance, *R. fuelleborni* shows slight constriction at the oesophageal apex (Müller et al., 2023); *R. stenocephala* exhibits a distinct constriction at the anterior body near the oesophageal apex (Kuzmin et al., 2016); and *R. megacephala* has shoulder-like expansions at the level of the nerve ring (Euclydes et al., 2024).

Host phylogeny is a key determinant of parasite community structure, as host evolutionary history influences parasite dispersal and host-switching (Poulin, 2007). Host traits, such as physiology, behaviour, and ecology, also facilitate parasite colonization (Rezende et al., 2009; Dormann et al., 2017; D’Bastiani et al., 2020; Tavares-Costa et al., 2022). Thus, the close phylogenetic relationship between the sister families Odontophrynidae and Bufonidae, both part of the monophyletic group Commutibirana (Hime et al., 2021; Portik et al., 2023), reflects the evolutionary proximity of their respective parasites. This suggests that the observed parasite subclade formation was likely influenced by host phylogeny.

Furthermore, it is important to highlight that Tkach and Snyder, (2014) proposed that multiple colonization events occurred throughout *Rhabdias* evolution, with host-switching being a recurrent feature. They suggested that host-switching and ecological adaptation provided more evolutionary advantages to *Rhabdias* spp. than the association with 1 host taxon. Thus, the observed zoogeographic patterns and the restriction to *Rhabdias* spp. to specific biogeographic regions are, in part, a result of these parasites’ abilities to successfully colonize new hosts as they become available in different areas.

Species differentiation/diversification in *Rhabdias* is a complex process influenced by a combination of mechanisms, including intrinsic and host-related processes (Tkach and Snyder, 2014; Müller et al., 2018, 2023). Some studies have explored these mechanisms using morphology, genetic, ecological adaptations, and parasite functional traits (Poulin, 2011; Kamiya et al., 2014). The observed patterns, so far, highlight that ecological fitting and host-switching are also key factors in the speciation ot lung-dwelling nematodes. These mechanisms are crucial for understanding the origin and diversification of parasite lineages in Neotropical amphibian hosts.

Fifteen nominal *Rhabdias* species are recognized in Brazilian anurans (Euclydes et al., 2024). However, considering the 5 known *Dendrobates* species and over 200 species in Dendrobatidae (Frost, 2025), the diversity of *Rhabdias* in these hosts remains poorly understood. To date, only 2 dendrobatid hosts have been recorded for *Rhabdias* spp.: *R. galactonoti* in *Adelphobates galactonotus* (Kuzmin et al., 2016) and *Rhabdias* sp. in *Ameerega pulchripecta* (Tavares-Costa et al., 2019). Thus, *Dendrobates tinctorius* represents the third dendrobatid host for *Rhabdias*.

The knowledge of *Rhabdias* diversity has advanced in recent years with new species descriptions, phylogeny studies, and biogeographic surveys (Müller et al., 2018; Tavares-Costa et al., 2022; Alcantara et al., 2023; Euclydes et al., 2024). It is important to highlight that most Brazilian species have been described in the Northern region, particularly in the Amazon. Our research group has described 7 species of the Rhabdiasidae to date (Santos et al., 2011; Nascimento et al., 2013; Kuzmin et al., 2016; Machado et al., 2018; Willkens et al., 2019; Tavares-Costa et al., 2022), using integrative studies of helminths from Amazonian amphibians and reptiles. Therefore, the predominance of known species in the Northern region may reflect a sampling bias, driven by intensive research efforts in this area and the presence of a core group of taxonomists dedicated exclusively to studying these parasites in amphibians and reptiles.

Until now, only 1 *Rhabdias* species has been formally described from a dendrobatid host, suggesting these associations are rare or have been historically underestimated. We propose 2 hypotheses for this gap: (1) a historical lack of parasitological surveys targeting Dendrobatidae; (2) potentially high host specificity in *Rhabdias,* limiting colonization of chemically defended hosts; and the possible inhibitory role of host alkaloids. Additionally, all known records were made in Brazil, which could reflect the emergence of new parasitology researchers in the area in the recent 20 years.

Dendrobatids, or poison frogs, are chemically defended Neotropical anurans that produce potent skin alkaloids (Daly et al., 2005; Saporito et al., 2012). These compounds are important modulators of skin microbiota and aid frogs to escape from predators (Johnson et al., 2018; Christian et al., 2021; Caty et al., 2024), however, it is still unclear how skin-penetrating parasites, such as *Rhabdias* spp., overcome this chemical barrier. This is particularly interesting, since dendrobatids hosts only a few species of *Rhabdias*, suggesting that these alkaloids may act as defense mechanisms for these parasites. Additionally, we still have little information on the excretory/secretory system and life cycle of *Rhabdias* spp. (Melo et al., 2016). The molecules produced by nematodes may act during skin penetration, leading to successfully infecting some hosts.

Considering that *Dendrobates* species secrete toxic skin alkaloids as a defense mechanism, infection by *Rhabdias* suggests potential parasite adaptations to overcome such chemically hostile environments, offering a promising model to study resistance to bioactive compounds. Thus, investigating these interactions will provide valuable insights into host–parasite coevolution and the ecological constraints shaping parasitic specificity in Neotropical systems.

The new species described herein represents the eighth *Rhabdias* species reported from the Amazon region and the first documented in a *Dendrobates* host. This finding adds new data to *Rhabdiasidae* diversity in Amazonian amphibians and raises new questions about parasite interactions with chemically defended anurans. Furthermore, given the ecological complexity and immense biological potential of the Amazon biome, additional studies integrating taxonomic, ecological, and physiological approaches are crucial to uncover the hidden diversity of nematodes parasitizing Neotropical amphibians.

## Supporting information

10.1017/S003118202510108X.sm001 Tavares-Costa et al. supplementary material Tavares-Costa et al. supplementary material

## Data Availability

Not applicable.

## Supplementary material

The supplementary material for this paper can be found at https://doi.org/10.1017/S003118202510108X.

## References

[ref1] Alcantara EP, Müller MI, Úngari LP, Ferreira-Silva C, Emmerich E, Giese EG, Morais DH, Santos ALQ, O’Dwyer LH and Silva RJ (2023) Integrative taxonomy in the genus *Rhabdias* Stiles et Hassall, 1905 from anuran in Brazil, description of two new species and phylogenetic analyses. *Parasitology International* 93, 102714. 10.1016/j.parint.2022.102714 36462634

[ref2] Bursey CR and Goldberg SR (2005) New species of *Oswaldocruzia* (Nematoda: Molineoidae), new species of *Rhabdias* (Nematoda: Rhabdiasidae), and other helminths in *Rana* cf. *forreri* (Anura: Ranidae) from Costa Rica. *Journal Parasitology* 91, 600–605. 10.1645/GE-3440 16108553

[ref3] Bursey CR, Goldberg SR and Telford SR (2003) *Rhabdias anolis* n. sp. (Nematoda: Rhabdiasidae) from the lizard, *Anolis frenatus* (Sauria: Polychrotidae), from Panama. *Journal Parasitology* 89, 113–117. 10.1645/0022-3395 12659312

[ref4] Bursey CR, Goldberg SR and Vitt LJ (2007) New species of *Rhabdias* (Nematoda: Rhabdiasidae) and other helminths from *Norops capito* (Sauria: Polychrotidae) from Nicaragua. *Journal Parasitology* 93, 29–131. 10.1645/GE-887R.1 17436951

[ref5] Bush AO, Laferty KD, Lotz JM and Shostak AW (1997) Parasitology meets ecology on its own terms: Margolis et al., revisited. *Journal of Parasitology* 83, 575–583. 9267395

[ref6] Caty SN, Alvarez-Buylla A, Vasek C, Tapia EE, Martin NA, McLaughlin T, Weber PK, Mayali X, Coloma LA, Morris MM and O’Connell LA (2024) A toxic environment selects for specialist microbiome in poison frogs. *bioRxiv* 10.1101/2024.01.10.574901

[ref7] CFMV (2013) Conselho Federal de Medicina Veterinária. Métodos de eutanásia. In *Guia Brasileiro de Boas Práticas de Eutanásia Em Animais. Comissão de Ética, Bioética E Bem-estar Animal*. CFMV. Brasília, Distrito Federal, 28–29.

[ref8] Christian K, Shine R, Day KA, Kaestli M, Gibb K, Shilton CM and Brown GP (2021) First line of defence: skin microbiota may protect anurans from infective larval lungworms. *International Journal for Parasitology: Parasites and Wildlife* 14, 185–189. 10.1016/j.ijppaw.2021.02.014 33898219 PMC8056135

[ref9] D’Bastiani E, Campião KM, Boeger WA and Araújo SB (2020) The role of ecological opportunity in shaping host–parasite networks. *Parasitology* 147(13), 1452–1460. 10.1017/S003118202000133X 32741380 PMC10317776

[ref10] Da Silva ICO, Soares P, Miguel MC, Couto RMP, Miranda GM, Alves AM and Paiva F (2019) First record of *Rhabdias* cf. *breviensis* (Rhabditoidea: Rhabdiasidae) parasitizing *Scinax acuminatus* (Anura: Hylidae) in the southern pantanal wetland, Brazil. *Herpetology Notes* 12, 975–980.

[ref11] Daly JW, Spande TF and Garraffo HM (2005) Alkaloids from amphibian skin: a tabulation of over eight-hundred compounds. *Journal of Natural Products* 68(10), 1556–1575. 10.1021/np0580560 16252926

[ref12] Dormann CF, Von RL and Scherer-Lorenzen M (2017) No consistent effect of plant species richness on resistance to simulated climate change for above- or below-ground processes in managed grasslands. *BMC Ecology* 17(23), 1–12. 10.1186/s12898-017-0133-0 28623883 PMC5473966

[ref13] Edgar RC (2004) Muscle: a multiple sequence alignment method with reduced time and space complexity. *BMC Bioinformatics* 5(113), 1–19. 10.1186/1471-2105-5-113 15318951 PMC517706

[ref14] Euclydes R, Melo FTV, da Justa Hc, Jesus RF, Gremski LH, Veiga SS and Campião KM (2024) A new species of lungworm from the Atlantic Forest: *Rhabdias megacephala* n. sp. parasite of the endemic anuran *Proceratophrys boiei*. *Journal of Helminthology* 98, e51. 10.1017/S0022149X24000385 39291544

[ref15] Frost DR (2025) Amphibian species of the world: An online reference. Version 6.1. New York, NY, USA, American Museum of Natural History. http://research.amnh.org/herpetology/amphibia/index.html (accessed 30 April 2025).

[ref16] Guindon S and Gascuel O (2003) A simple, fast, and accurate algorithm to estimate large phylogenies by maximum likelihood. *Systematic Biology* 52, 696–704. 10.1080/10635150390235520 14530136

[ref17] Hime PM, Lemmon AR, Lemmon ECM, Prendini E, Brown JM, Thomson RC, Kratovil JD, Noonan BP, Pyron RA, Peloso PLV, Kortyna ML, Keogh JS, Donnellan SC, Mueller RL, Raxworthy CJ, Kunte K, Ron SR, Das S, Gaitonde N, Green DM, Labisko J, Che J and Weisrock DW (2021) Phylogenomics reveals ancient gene tree discordance in the amphibian tree of life. *Systematic Biology* 70(1), 49–66. 10.1093/sysbio/syaa034 32359157 PMC7823230

[ref18] Johnson PT, Calhoun DM, Stokes AN, Susbilla CB, McDevitt‐Galles T, Briggs CJ, Hoverman JT, Tkach VV and Roode JC (2018) Of poisons and parasites – the defensive role of tetrodotoxin against infections in newts. *Journal of Animal Ecology* 87(4), 1192–1204. 10.1111/1365-2656.12816 29476541

[ref19] Kamiya T, O’Dwyer K, Nakagawa S and Poulin R (2014) What determines species richness of parasitic organisms? A meta-analysis across animal, plant and fungal hosts: determinants of parasite species richness. *Biological Reviews* 89, 123–134. 10.1111/brv.12046 23782597

[ref20] Kearse M, Moir R, Wilson A, Stones-Havas S, Cheung M, Sturrock S and Drummond A (2012) Geneious Basic: an integrated and extendable desktop software platform for the organization and analysis of sequence data. *Bioinformatics* 28, 1647–1649. 10.1093/bioinformatics/bts199 22543367 PMC3371832

[ref21] Kimura M (1980) A simple method for estimating evolutionary rate of base substitutions through comparative studies of nucleotide sequences. *Journal of Molecular Evolution* 16, 111–120. 7463489 10.1007/BF01731581

[ref22] Kloss GR (1971) Alguns *Rhabdias* (Nematoda) de *Bufo* no Brasil. *Papéis Avulsos do Departamento de Zoologia de São Paulo* 24(1), 1–52.

[ref23] Kloss GR (1974) *Rhabdias* (Nematoda, Rhabditoidea) from the marinus group of Bufo. A study of sibling species. *Arq Zool* 25, 61–120. 10.11606/issn.2176-7793.v25i2p61-120

[ref24] Kuzmin Y (2013) Review of Rhabdiasidae (Nematoda) from the Holarctic. *Zootaxa* 3639(1), 1–76. 10.11646/zootaxa.3639.1.1 25325086

[ref25] Kuzmin Y, Du Preez L and Junker K (2015) Some nematodes of the genus *Rhabdias* Stiles et Hassall, 1905 (Nematoda: Rhabdiasidae) parasitising amphibians in French Guine. *Folia Parasitologica* 62, 031. 10.14411/fp.2015.031 26084336

[ref26] Kuzmin Y, Melo FTV, da Silva Filho HF and Santos JN (2016) Two new species of *Rhabdias* Stiles et Hassall, 1905 (Nematoda: Rhabdiasidae) from anuran amphibians in Pará, Brazil. *Folia Parasitologica* 63(015), 1–10. 10.14411/fp.2016.015 27189518

[ref27] Kuzmin Y, Svitin R, McAllister CT, Guderyahn L and Tkach VV (2024) Descriptions and phylogenetic affinities of two new species of *rhabdias* stiles and Hassall, 1905 (Nematoda: Rhabdiasidae) from North American Frogs: are we still only scratching the surface? *The Journal of Parasitology* 110(4), 339–350. 10.1645/24-10 39099080

[ref28] Kuzmin Y and Tkach VV (2025) List of species. *Rhabdias*. The nematode family Rhabdiasidae http://izan.kiev.ua/ppages/rhabdias (accessed 26 June 2025).

[ref29] Kuzmin Y, Tkach VV and Brooks DR (2007) *Rhabdias alabialis* sp. nov. and *R. pseudosphaerocephala* sp. nov. (Nematoda: Rhabdiasidae) in the marine toad, *Bufo marinus* (L.) (Lissamphibia: Anura: Bufonidae) in central America. *The Journal of Parasitology* 93, 159–165. 10.1645/GE-858R.1 17436957

[ref30] Langford GJ and Janovy J (2013) Host specificity of North American *Rhabdias* spp. (Nematoda: Rhabdiasidae): combining field data and experimental infections with a molecular phylogeny. *Journal of Parasitology* 99, 277–286. 10.1645/GE-3217.1 22988815

[ref31] Machado SA, Kuzmin Y, Tkach V, Santos JN, Gonçalves EC and Melo FTV (2018) Description, biology and molecular characterisation of *serpentirhabdias moi* n. sp.(Nematoda: Rhabdiasidae) from *chironius exoletus* (Serpentes: Colubridae) in Brazil. *Parasitology International* 67, 829–837. 10.1016/j.parint.2018.05.004 29753096

[ref32] Martinez-Salazar EA (2006) A new rhabdiasid species from *norops megapholidotus* (Sauria: Polychrotidae) from Mexico. *Journal Parasitology* 92, 325–1329. 10.1645/GE-872R1.1 17304815

[ref33] Martinez-Salazar EA (2008) A new rhabdiasid species from *Craugastor occidentalis* (Anura: Brachycephalidae) from Sierra de Manantlán Jalisco, Mexico. *Revista Mexicana de Biodiversidad* 79, 81–89.

[ref34] Martinez-Salazar EA and Leon-Regagnon V (2007) New species of *Rhabdias* (Nematoda: Rhabdiasidae) from *bufo occidentalis* (Anura: Bufonidae) from Sierra Madre del Sur, Mexico. *Journal Parasitology* 93, 1171–1177. 10.1645/GE-1188R.1 18163354

[ref35] Melo FTV, Nascimento LCS, Macedo LC, Santos JN and Kuzmin Y (2016) The morphology of free-living stages and immature parasites of *Rhabdias paraensis* (Nematoda: Rhabdiasidae), a parasite of *Rhinella marina* (Anura: Bufonidae) in Brazil. *Acta Parasitologica* 61, 42–51. 10.1515/ap-2016-0004 26751870

[ref36] Miller MA, Pfeifer W and Schwartz T (2010) Creating the CIPRES Science Gateway for inference of large phylogenetic trees in Proceedings of the Gateway computing environments workshop (GCE), November 2010. New Orleans, LA.

[ref37] Morais DH, Müller MI, Melo FTV, Aguiar A, Willkens Y, De Sousa SC, Giese EG, Ávila RW and da Silva RJ (2020) A new species of *Rhabdias* (Nematoda: Rhabdiasidae), a lung parasite of *Pseudopaludicola pocoto* (Anura: Leptodactylidae) from north-eastern Brazil: Description and phylogenetic analyses. *Journal of Helminthology* 94, 1–11. 10.1017/S0022149X20000929 33138887

[ref38] Moravec F and Kaiser H (1995) Helminth parasites from west Indian frogs, with descriptions of two new species, Caribb. *Journal of Science* 31, 252–268.

[ref39] Moravec F and Sey O (1990) Some nematode parasites of frogs from Papua New Guinea and Australia. *Acta Societatis Zoologicae Bohemicae* 54, 268–286.

[ref40] Moskowitz NA, D’Agui R and O’Connell LA (2022) Poison frog dietary preference depends on prey type and alkaloid load. *bioRxiv* 1, 1–28. 10.1371/journal.pone.0276331 PMC9714857 36454945

[ref41] Müller MI, Morais DH, Costa-Silva GJ, Aguiar A, Ávila RW and da Silva RJ (2018) Diversity in the genus *Rhabdias* (Nematoda, Rhabdiasidae): evidence for cryptic speciation. *Zoologica Scripta* 47, 595–607. 10.1111/zsc.12304

[ref42] Müller MI, Morais DH, Tavares-Costa LFS, de Vasconcelos Melo FT, Giese EG, Ávila RW and da Silva RJ (2023) Revisiting the taxonomy of *Rhabdias fuelleborni* travassos, 1928 (Nematoda, Rhabdiasidae) with approaches to delimitation of species and notes on molecular phylogeny. *Parasitology International* 92, 102692. 10.1016/j.parint.2022.102692 36341837

[ref43] Nascimento LDCS, Gonçalves EC, Melo FTV, Giese EG, Furtado AP and Santos JN (2013) Description of *Rhabdias breviensis* n. sp. (Rhabditoidea: Rhabdiasidae) in two neotropical frog species. *Systematic Parasitology* 86(1), 69–75. 10.1007/s11230-013-9432-9 23949651

[ref44] Pleijel F, Jondelius U, Norlinder E, Nygren A, Oxelman B, Schander C, Sundberg P and Thollesson M (2008) Phylogenies without roots? A plea for the use of vouchers in molecular phylogenetic studies. *Molecular Phylogenetics & Evolution* 48, 369–371. 10.1016/j.ympev.2008.03.024 18424089

[ref45] Portik DM, Streicher JW and Wiens JJ (2023) Frog phylogeny: a time-calibrated, species-level tree based on hundreds of loci and 5,242 species. *Molecular Phylogenetics & Evolution* 188, 107907. 10.1016/j.ympev.2023.107907 37633542

[ref46] Posada D (2008) jModelTest: phylogenetic model averaging. *Molecular Biology and Evolution* 25, 1253–1256. 10.1093/molbev/msn083 18397919

[ref47] Poulin R (2007) Are there general laws in parasite ecology? *Parasitology* 134, 763–776. 10.1017/S0031182006002150 17234043

[ref48] Poulin R (2011) Host specificity in phylogenetic and geographic space. *Trends in Parasitology* 27, 355–361. 10.1016/j.pt.2011.05.003 21680245

[ref49] Rambaut A (2009) Fig Tree v1.3.1. Institute of Evolutionary Biology, University of Edinburgh. Available at http://tree.bio.ed.ac.uk/software/figtree/ (accessed 09 April 2025).

[ref50] Rezende EL, Albert EM, Fortuna MA and Bascompte J (2009) Compartments in a marine food web associated with phylogeny, body mass, and habitat structure. *Ecology Letters* 12(8), 779–788. 10.1111/j.1461-0248.2009.01327.x 19490028

[ref51] Rojas B and Pašukonis A (2019) From habitat use to social behavior: Natural history of a voiceless poison frog, *Dendrobates tinctorius*. *PeerJ* 7, e7648. 10.7717/peerj.7648 31576237 PMC6753930

[ref52] Ronquist F, Huelsenbeck JP, Teslenko M, Zhang C and Nylander JA (2003) MrBayes version 3.2 manual: tutorials and model summaries. Available at https://nbisweden.github.io/MrBayes/download.html (accessed 30 April 2025).

[ref53] Santos JN, Melo FTV, Nascimento LCS, Nascimento DEB, Giese EG and Furtado AP (2011) *Rhabdias paraensis* sp. nov: a parasite of the lungs of *Rhinella marina* (Amphibia: Bufonidae) from Brazilian Amazonia. *Mem. Inst. Oswaldo Cruz* 106, 433–440. 10.1590/S0074-0276201100040 21739030

[ref54] Saporito RA, Donnelly MA, Spande TF and Garraffo HM (2012) A review of chemical ecology in poison frogs. *Chemoecology* 22, 159–168. 10.1007/s00049-011-0088-0

[ref55] Tamura K, Stecher G and Kumar S (2021) MEGA11: molecular evolutionary genetics analysis version 11. *Molecular Biology and Evolution* 38, 3022–3027. 10.1093/molbev/msab120 33892491 PMC8233496

[ref56] Tavares-Costa LFS, Dias-Souza MR, Costa-Campos CE and Melo FTV (2019) Helminth parasites of *Ameerega pulchripecta* (Anura: Dendrobatidae) from the eastern Amazon, Brazil. *Herpetology Notes* 12, 435–437.

[ref57] Tavares-Costa LFS, Rebêlo GL, Müller MI, Jesus RF, Nandyara B, Silva LMO, Costa-Campos CE, Santos JN and Melo FTV (2022) A new species of *Rhabdias* (Nematoda: Rhabdiasidae), a lung parasite of *Pristimantis chiastonotus* (Anura: Strabomantidae) from the Brazilian Amazon: description and phylogenetic analyses. *Parasitology Research* 121, 155–166. 10.1007/s00436-021-07396-1 34993630

[ref58] Tkach VV, Kuzmin Y and Snyder SD (2014) Molecular insight into systematics, host associations, life cycles and geographic distribution of the nematode family Rhabdiasidae. *International Journal for Parasitology* 44, 273–284. 10.1016/j.ijpara.2013.12.005 24560917

[ref59] Travassos L (1926) Entwicklung des *Rhabdias fuelleborni* n. sp. *Deutsche Tropenmedizinische Zeitschrift* 30, 594–602.

[ref60] Willkens Y, Rebêlo GL, Santos JN, Furtado AP, Vilela RV, Tkach VV, Kuzmin Y and Melo FTV (2019) *Rhabdias glaurungi* sp. nov. (Nematoda: Rhabdiasidae), parasite of *Scinax* gr. *ruber* (Laurenti, 1768) (Anura: Hylidae), from the Brazilian Amazon. *Journal of Helminthology* 94, e54. 10.1017/S0022149X19000476 31630693

[ref61] Xia X (2018) DAMBE7: new and improved tools for data analysis in molecular biology and evolution. *Molecular Biology and Evolution* 35, 1550–1552. 10.1093/molbev/msy073 29669107 PMC5967572

